# The association of wildfire smoke with respiratory and cardiovascular emergency department visits in Colorado in 2012: a case crossover study

**DOI:** 10.1186/s12940-016-0146-8

**Published:** 2016-06-04

**Authors:** Breanna L. Alman, Gabriele Pfister, Hua Hao, Jennifer Stowell, Xuefei Hu, Yang Liu, Matthew J. Strickland

**Affiliations:** The Office of Air Quality Planning and Standards, United States Environmental Protection Agency, 109 T.W Alexander Dr, Research Triangle Park, NC 27711 USA; National Center for Atmospheric Research, 3450 Mitchell Lane, Boulder, CO 80301 USA; Department of Environmental Health, Rollins School of Public Health, Emory University, 1518 Clifton Rd NE, Atlanta, GA 30322 USA; School of Community Health Sciences, University of Nevada, Reno, 1664 North Virginia Street, Reno, NV 89557 USA

**Keywords:** Wildfires, Respiratory, Cardiovascular, Emergency department visits, PM2.5, Particulate matter

## Abstract

**Background:**

In 2012, Colorado experienced one of its worst wildfire seasons of the past decade. The goal of this study was to investigate the relationship of local PM_2.5_ levels, modeled using the Weather Research and Forecasting Model with Chemistry, with emergency department visits and acute hospitalizations for respiratory and cardiovascular outcomes during the 2012 Colorado wildfires.

**Methods:**

Conditional logistic regression was used to assess the relationship between both continuous and categorical PM_2.5_ and emergency department visits during the wildfire period, from June 5^th^ to July 6^th^ 2012.

**Results:**

For respiratory outcomes, we observed positive relationships between lag 0 PM_2.5_ and asthma/wheeze (1 h max OR 1.01, 95 % CI (1.00, 1.01) per 10 μg/m^3^; 24 h mean OR 1.04 95 % CI (1.02, 1.06) per 5 μg/m^3^), and COPD (1 h max OR 1.01 95 % CI (1.00, 1.02) per 10 μg/m^3^; 24 h mean OR 1.05 95 % CI (1.02, 1.08) per 5 μg/m^3^). These associations were also positive for 2-day and 3-day moving average lag periods. When PM_2.5_ was modeled as a categorical variable, bronchitis also showed elevated effect estimates over the referent groups for lag 0 24 h average concentration. Cardiovascular results were consistent with no association.

**Conclusions:**

We observed positive associations between PM_2.5_ from wildfire and respiratory diseases, supporting evidence from previous research that wildfire PM_2.5_ is an important source for adverse respiratory health outcomes.

**Electronic supplementary material:**

The online version of this article (doi:10.1186/s12940-016-0146-8) contains supplementary material, which is available to authorized users.

## Background

Between March 26^th^ and July 10^th^ 2012, Colorado experienced one of its worst wildfire seasons of the past decade [1]. By the time the final fires were contained, over 600 homes had been destroyed [2], and an estimated 32,000 people had been evacuated from areas near actively burning fires [3]. While the physical damage to homes and property is readily apparent, wildfire smoke is also a health hazard.

Concentrations of particulate matter less than 2.5 μm in diameter (PM_2.5_) can be substantially elevated during wildfire events compared to non-fire situations [4]. Short-term increases in outdoor PM_2.5_ concentrations have been designated as “likely to be causal” in regard to respiratory morbidity and “causal” in regard to cardiovascular morbidity by the US Environmental Protection Agency, based on current epidemiologic and toxicological literature [5], and wildfire PM_2.5_ has been linked with several health problems, most notably adverse respiratory outcomes [6]. Recent toxicological studies have shown that PM_2.5_ from wildfires may have different health effects than typical urban ambient PM_2.5_, particularly in the amount of oxidative stress generated, which may be due to differences in chemical composition [7, 8].

Although many epidemiologic studies have examined associations between respiratory and cardiovascular hospital admissions and urban air pollution, studies on the health effects of wildfire smoke are less common. Associations have been reported between exposure to PM_2.5_ during wildfires and hospital admissions [9] and emergency department (ED) visits [10–14] for respiratory illnesses. Cardiovascular morbidity has been linked to exposure to ambient PM [5], but among PM_2.5_ and PM_10_ from wildfires, results have been less consistent, with some studies showing a positive relationship [9, 10, 14], some showing negative relationships [15] and some showing no relationship [13, 16–18].

The 2012 Colorado wildfires present an interesting situation: they burned continuously throughout the summer months, affected a wide geographic area across the state, thus allowing for a larger sample size than often found during typical wildfire periods, and created highly variable PM_2.5_ concentrations both spatially and temporally. Given the intensity of the Colorado wildfire season of 2012 and the potential for strong adverse respiratory effects from exposure to particulate matter from wildfires, it is important to assess the health impacts associated with wildfire air pollution. The goal of this study is to estimate associations between local PM_2.5_ levels and ED visits and acute hospitalizations for six respiratory and seven cardiovascular outcomes during the Colorado wildfires of 2012.

## Methods

Hourly PM_2.5_ concentrations between June 5th and July 6th 2012 were modeled using the Weather Research and Forecasting Model with Chemistry (WRF-Chem) [19]. This model was run at a 12 km by 12 km spatial resolution across the Western US, and its outputs were used to characterize PM_2.5_ and ozone throughout Colorado. The Model for Ozone and Related chemical Tracers (MOZART-4) [20] was used for the chemical boundary conditions, and the National Center for Environmental Protection’s North American Mesoscale Forecast System (NCEP/NAM) was used for the meteorological boundary conditions. The wildfire emission estimates used to inform the model are based on the NCAR Fire Inventory (FINN) [21] with the burned area product from the SMARTFIRE framework (provided by Sean Raffuse, Sonoma Technology). The model simulations have been evaluated with operational meteorological observations, satellite retrievals of carbon monoxide, aerosol optical depth, ozone, and PM_2.5_ measurements from the EPA surface network. The latter are most relevant when using the model product in a health analysis, though it cannot be expected that the often highly localized characteristics of these sites can be captured by the model’s spatial resolution. For these analyses, we calculated the PM_2.5_ 24 h daily mean and 1 h daily maximum for each of these 12 km by 12 km grid cells. Fine particles were identified as the size of interest because of their overall association with cardiovascular and respiratory endpoints [5]. Although 24 h average PM_2.5_ concentrations may be the most relevant index for health effects [5], 1 h max concentrations were also assessed, as a high 1 h max concentration could trigger a health event. While the fires burned between March 26^th^ and July 10^th^, WRF-Chem simulation was conducted only for the peak burning period from June 5^th^ to July 6^th^.

Temperature data in 12 km by 12 km grid cells were interpolated from the North American Land Data Assimilation System (NLDAS) output at ~14 km resolution [22]. These data are included as a covariate in the epidemiologic analysis, using the mean recorded temperature for the day within the specified area. Grid-level exposure was estimated by spatially joining the meteorological data with the health data.

Staff at the Colorado Department of Public Health and Environment geocoded patient addresses for hospitalizations and ED visits for cardiorespiratory disease during June 5^th^ 2012 to July 6^th^ 2012 to 12 km grids. Data elements include information on the age, sex, date of admission, the International Classification of Diseases version 9 (ICD9) code, and payment method of the patient, and are expected to capture all ED visits and hospitalizations in Colorado. Patients living in Colorado with addresses that could not be geocoded were excluded (870 of 10,699 records (8.1 %) could not be geocoded). This study population included patients of all ages.

We examined six respiratory and seven cardiovascular endpoints. Cases were identified using the primary International Classification of Diseases version 9 (ICD 9) diagnosis code. The respiratory endpoints were upper respiratory disease (ICD9:460–465, 466.0), pneumonia (ICD9: 480–486), bronchitis (ICD9: 490), chronic obstructive pulmonary disease (COPD) (ICD9: 491, 492, 496), asthma and wheeze (ICD9: 493–786.07), and respiratory disease (ICD9: 460–465, 466.0, 466.1, 466.11, 466.19, 480–486, 487, 488, 490, 491, 492, 496, 493–786.07). The cardiovascular endpoints were acute myocardial infarction (MI) (ICD9: 410), ischemic heart disease (IHD) (ICD9: 410–414), dysrhythmia (ICD9: 427), congestive heart failure (ICD9: 428) (CHF), ischemic stroke (ICD9: 433–437), peripheral vascular disease (ICD9: 440, 443, 444, 451–453), and cardiovascular disease (CVD) (ICD9: 410–414, 427, 428, 433–437, 440, 443, 444, 451–453). We analyzed all ED visits and all hospitalizations, with the exception of patient hospitalizations that had an “elective” admit type code and patients who were hospitalized because they came through the emergency room, to avoid double counting. All ED visits and hospitalizations included in the analysis will hereafter be referred to as “ED visits”. Due to the lack of individual identifiers, patients that had multiple ED visits were counted multiple times. Human subjects research approved by Emory University IRB #00066505.

Conditional logistic regression, where each grid was matched to itself over the 32-day study period, was used to estimate associations between PM_2.5_ concentrations and the occurrence of ED and acute hospitalizations for each endpoint. This approach compares the number of cases on each day with the number of cases on the other days within the same grid and is analogous to time series study analyzed using conditional logistic regression to control for grid location or a case-crossover analysis with pooling across days in a given stratum [23]. By stratifying on grid cell, this approach controls for time-invariant confounders that vary spatially but not those confounders that vary temporally.

PM_2.5_ was modeled as both a continuous and categorical variable to investigate possible departures from linearity. We controlled for grid-level day-of temperature and day of week, and daily 8-h maximum ozone was assessed as a potential confounder in two-pollutant models, but was dropped for parsimony. The analyses spanned from June 5, 2012 to July 6, 2012, thus resulting in 32 observations per stratum. Concordant strata (i.e., those with zero ED visits during the 32-day period) were dropped from the analysis. There were no missing exposure data. All analyses were conducted in SAS 9.3 (Cary, NC). Three different lag periods were examined, lag 0, lag 0–1 moving average, and lag 0-1-2 moving average. We stratified asthma and respiratory disease results by age to examine effect modification.

## Results

**Fig. 1:**
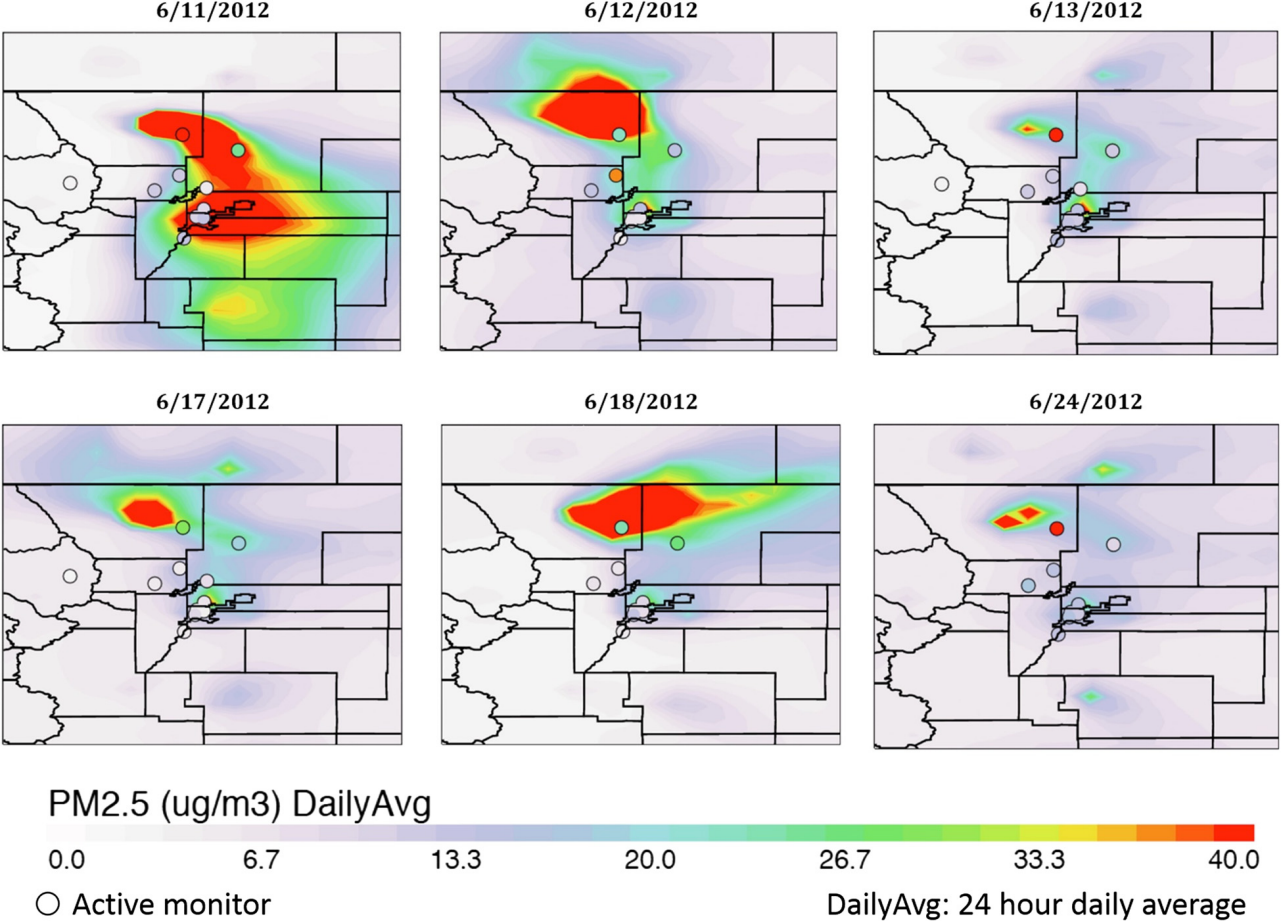
Spatial variability of PM_2.5_ 24 h average concentrations from June 11^th^ to June 24^th^

PM_2.5_ model data for the study period are presented in Fig. 1, which shows the location of monitors within the Denver area at six points throughout the wildfire period, as well as a comparison of their measured values to the modelled data. Gridded air quality surfaces were smoothed to better see how variable the plumes were throughout the study period. A more comprehensive comparison of modelled vs monitor data at 21 sites throughout Colorado is available in [Additional file 1: Figure S1], which shows that accuracy to monitor data varied both temporally and spatially. Overall, the model had an absolute bias (i.e., a directionless measure of the average difference between the measured and modelled estimations) of 13 μg/m^3^ for PM_2.5_ concentrations for the 6 stations in and around the Denver Metro Area, an area with little fire impact, 13 μg/m^3^ for the 2 stations north-east of Denver, the area most impacted by fires, and 19 μg/m^3^ for the station east of Denver compared to the monitor data. 1-h maximum PM_2.5_ ranged from 2.02 μg/m^3^ to greater than 5000 μg/m^3^ during the study period. As expected, the more extreme model-simulated PM_2.5_ levels took place on days of intensive fire activity and at locations near active fire sites. Additionally, PM_2.5_ levels showed a clear diurnal trend, with an increase in the late afternoon (~4 pm) with peak levels around 7 pm and continuing elevated levels well into the late evening hours. In addition to fire activities, the collapsing planetary boundary layer in the evening would limit the convection of smoke plumes, therefore also contributing to these extreme concentrations during our study period. Moreover, this trend coincides with the diurnal fire emission profiles provided by the Western Regional Air Partnership (WARP) [24].

**Fig. 2:**
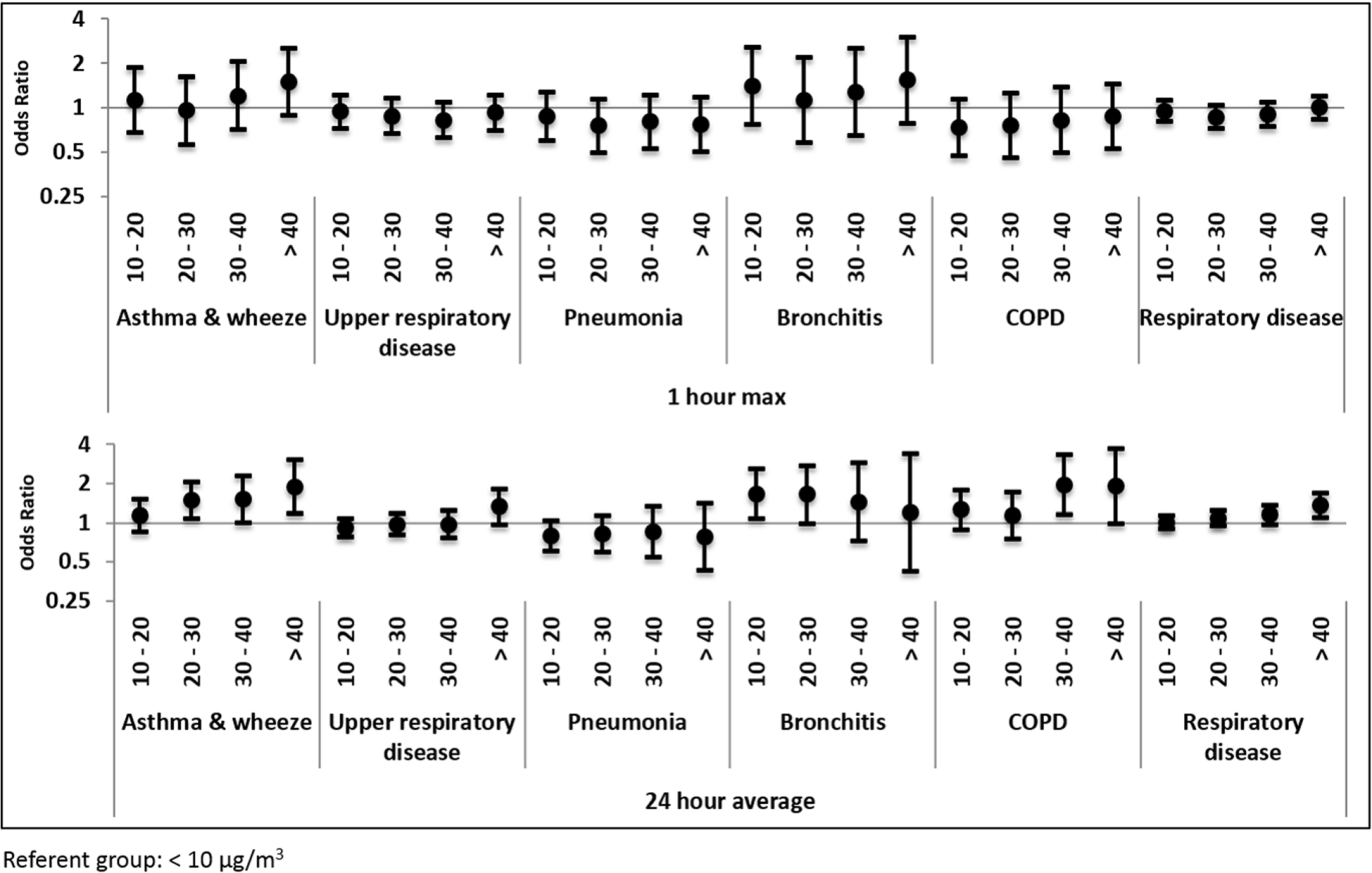
Concentration response odds ratios for respiratory outcomes for 24-h and 1-h maximum categorical PM_2.5_

**Fig. 3:**
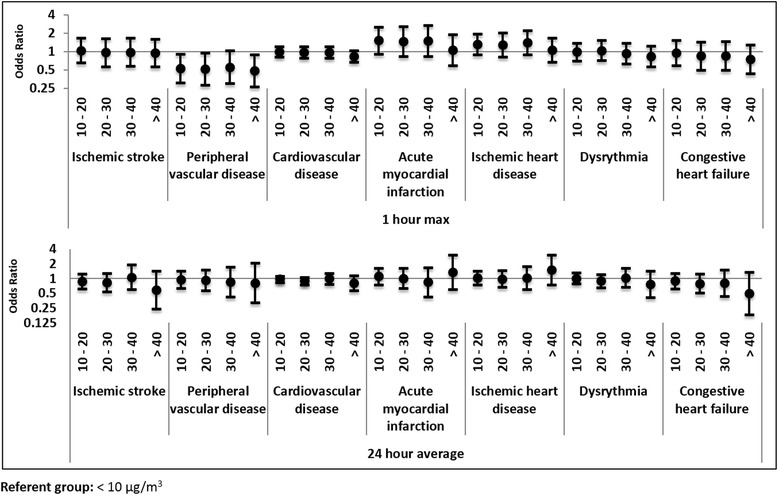
Concentration response odds ratios for cardiovascular outcomes for 24-h and 1-h maximum categorical PM_2.5_

For respiratory outcomes, when PM_2.5_ was examined as a continuous variable, we observed positive relationships between PM_2.5_ and asthma and wheeze (1 h max OR 1.01, 95 % CI (1.00, 1.01) per 10 μg/m^3^; 24 h mean OR 1.04 95 % CI (1.02, 1.06) per 5 μg/m^3^), and COPD (1 h max OR 1.01 95 % CI (1.00, 1.02) per 10 μg/m^3^; 24 h mean OR 1.05 95 % CI (1.02, 1.08) per 5 μg/m^3^) (Tables 1 and 2). PM_2.5_ was also examined as a categorical variable, with each category representing a 10 μg/m^3^ increase in PM_2.5_ concentration (Figs. 2 and 3). Positive associations between asthma and wheeze and 24 h mean PM_2.5_ were also observed when looked at over longer lag periods (Table 1). For case count per concentration category, see [Additional file 2: Table S2].

**Table 1:** Odds Ratios for respiratory and cardiovascular endpoints for continuous change in 24-h PM_2.5_ concentrations

Health endpoint	Case count	24 h mean OR^a^ (% change)		
		Lag 0	Lag 0-1^b^	Lag 0-1-2^b^
Respiratory				
Asthma & Wheeze (All ages)	1136	1.04 (1.02, 1.06)	1.05 (1.03, 1.07)	1.07 (1.04, 1.10)
(Ages 0–18)	387	1.02 (0.98, 1.07)	1.03 (0.96, 1.10)	1.05 (0.97, 1.14)
(Ages 19–64)	665	1.03 (1.00, 1.06)	1.03 (1.00, 1.06)	1.07 (1.02, 1.11)
(Ages 65+)	84	1.12 (0.99, 1.27)	1.30 (1.06, 1.60)	1.29 (1.03, 1.61)
Upper respiratory infection	3376	1.02 (1.00, 1.03)	1.01 (0.99, 1.04)	1.01 (0.98, 1.04)
Pneumonia	955	0.99 (0.95, 1.03)	1.00 (0.95, 1.04)	1.00 (0.95, 1.05)
Bronchitis	413	1.00 (0.94, 1.06)	1.01 (0.95, 1.08)	1.02 (0.94, 1.10)
COPD	628	1.05 (1.02, 1.08)	1.07 (1.03, 1.11)	1.07 (1.02, 1.12)
Respiratory disease (All ages)	6610	1.02 (1.01, 1.03)	1.03 (1.01, 1.04)	1.03 (1.02, 1.05)
(Ages 0–18)	2710	0.99 (0.97, 1.02)	0.99 (0.96, 1.02)	1.01 (0.97, 1.05)
(Ages 19–64)	2915	1.03 (1.01, 1.04)	1.03 (1.01, 1.05)	1.03 (1.01, 1.06)
(Ages 65+)	985	1.02 (0.98, 1.06)	1.03 (0.98, 1.09)	1.04 (0.98, 1.10)
Cardiovascular				
Acute myocardial infarction	462	1.00 (0.96, 1.05)	1.01 (0.96, 1.06)	1.00 (0.94, 1.06)
Ischemic heart disease	722	1.01 (0.98, 1.05)	1.03 (1.00, 1.08)	1.04 (1.00, 1.08)
Dysrhythmia	1000	0.97 (0.93, 1.02)	0.97 (0.92, 1.02)	0.92 (0.87, 0.98)
Congestive heart failure	510	0.93 (0.86, 1.00)	0.95 (0.88, 1.03)	0.94 (0.86, 1.03)
Ischemic Stroke	576	0.99 (0.95, 1.04)	0.96 (0.89, 1.03)	0.97 (0.90, 1.05)
Peripheral vascular disease	411	0.95 (0.87, 1.03)	0.97 (0.89, 1.06)	0.93 (0.84, 1.03)
Cardiovascular disease	3219	0.98 (0.96, 1.01)	0.98 (0.96, 1.01)	0.97 (0.94, 1.00)

^a^ Change per 5 μg/m^3^

^b^ Moving average

**Table 2:** Odds Ratios for respiratory and cardiovascular endpoints for continuous change in 24-h PM_2.5_ concentrations

Health endpoint	Case count	1 h max OR^a^		
		Lag 0	Lag 0-1^b^	Lag 0-1-2^b^
Respiratory				
Asthma & Wheeze (All ages)	1136	1.01 (1.00, 1.01)	1.07 (1.04, 1.10)	1.02 (1.01, 1.03)
(Ages 0–18)	387	1.00 (0.99, 1.02)	1.01 (0.99, 1.03)	1.02 (0.99, 1.05)
(Ages 19–64)	665	1.01 (1.00, 1.02)	1.01 (1.00, 1.01)	1.02 (1.00, 1.03)
(Ages 65+)	84	1.06 (1.00, 1.12)	1.09 (1.01, 1.18)	1.08 (1.00, 1.17)
Upper respiratory infection	3376	1.00 (1.00, 1.01)	1.03 (1.00, 1.01)	1.00 (0.99, 1.01)
Pneumonia	955	1.00 (0.99, 1.01)	1.00 (0.95, 1.05)	0.99 (0.98, 1.02)
Bronchitis	413	1.00 (0.98, 1.02)	1.00 (0.98, 1.03)	1.01 (0.98, 1.03)
COPD	628	1.01 (1.00, 1.02)	1.02 (1.01, 1.03)	1.02 (1.01, 1.04)
Respiratory disease (All ages)	6610	1.01 (1.00, 1.01)	1.01 (1.00, 1.01)	1.01 (1.00, 1.01)
(Ages 0–18)	2710	1.00 (0.99, 1.01)	1.00 (0.98, 1.01)	1.00 (0.99, 1.01)
(Ages 19–64)	2915	1.01 (1.00, 1.02)	1.01 (1.00, 1.01)	1.01 (1.00, 1.02)
(Ages 65+)	985	1.00 (0.99, 1.02)	1.01 (0.99, 1.03)	1.01 (0.99, 1.03)
Cardiovascular				
Acute myocardial infarction	462	1.00 (0.99, 1.02)	1.00 (0.98, 1.02)	1.01 (0.99, 1.02)
Ischemic heart disease	722	1.00 (0.99, 1.01)	1.03 (0.99, 1.02)	1.01 (0.99, 1.02)
Dysrhythmia	1000	0.98 (0.96, 1.00)	0.98 (0.96, 1.00)	0.97 (0.94, 0.99)
Congestive heart failure	510	0.98 (0.95, 1.01)	0.98 (0.95, 1.01)	0.97 (0.94, 1.01)
Ischemic Stroke	576	1.00 (0.99, 1.01)	0.99 (0.97, 1.02)	0.99 (0.96, 1.02)
Peripheral vascular disease	411	0.98 (0.95, 1.02)	0.98 (0.95, 1.02)	0.97 (0.93, 1.02)
Cardiovascular disease	3219	0.99 (0.98, 1.00)	0.99 (0.98, 1.01)	0.99 (0.98, 1.00)

^a^ Change per 10 μg/m^3^

^b^ Moving average

For the asthma and wheeze outcomes, we observed positive relationships with both 1 h max and 24 h average windows with increasing PM_2.5_ concentrations; this relationship was more pronounced in the 24 h analysis (Fig. 2). Bronchitis showed elevated effect estimates over the referent groups for 24 h average; however, the confidence intervals are generally wide, and the relationship with PM_2.5_ is suggestive of a plateau effect rather than a linear concentration-response relationship, with all effect estimates being similarly elevated over the referent group (<10 μg/m^3^), with the exception of the >40 μg/m^3^ group. COPD shows a similar pattern for 24 h average PM_2.5_, with all concentration groups effect estimates being elevated over the referent group, with the 30–40 ug/m^3^ (OR 1.97 95 % CI (1.16, 3.34)) and > 40 μg/m^3^ (OR 1.93 95 % CI (0.99, 3.74)) exposure categories being elevated over the 10–20 and 20–30 μg/m^3^ exposure groups (OR 1.27 95 % CI (0.90, 1.79) and OR 1.15 95 % CI (0.76, 1.74) respectively). However, these elevated associations lacked precision, and were not present in the 1 h max analyses. We also completed age-stratified analyses for two outcomes, asthma and respiratory disease. Overall, the highest magnitude of effect was seen in those aged 65 and up; however, results were imprecise (Tables 1 and 2).

For cardiovascular outcomes, point estimates were consistently null in the continuous analysis. Cardiovascular outcomes were also largely null in the categorical analysis (Fig. 3). There is some evidence for a negative association between PM_2.5_ and peripheral vascular disease for the 1 h daily max at lag 0, with the 10–20 μg/m^3^, 20–30 μg/m^3^, and >40 μg/m^3^ concentration group ORs falling below the null with similar magnitudes. CHF also had negative effect estimates for the continuous results across all lag periods. Acute myocardial infarction point estimates were all elevated above the null for the 1 h daily max exposure, with the exception of the highest exposure category (>40 μg/m^3^), though confidence intervals were wide and crossed the null.

## Discussion

The positive associations between PM_2.5_ from wildfires and respiratory disease observed in this study are consistent with previous published wildfire research. Rappold, et al. [10] found associations between asthma ED visits and wildfire smoke in North Carolina, with the strongest association observed at lag 0. This study averaged exposure to the county level, giving everyone within the county a uniform distribution of exposure. Rappold, et al. [14] and Moore, et al. [13] and also found positive associations between asthma exacerbation and PM_2.5_, looking at emergency department and physician visits respectively. We also observed similar positive associations with bronchitis and COPD as Rappold, et al. [14], although they looked specifically at acute bronchitis and pneumonia, whereas our study focused on bronchitis alone and found no association with pneumonia.

We observed differences in effect estimates with age, specifically those above 65 years of age had higher odds of an event, potentially identifying that age group as a susceptible population. Previous studies have also shown that young age can be a significant effect measure modifier with respiratory morbidity [25, 26]; however, when we stratified by age, effect estimates did not appear to be higher for those ages 0–18.

Overall, the cardiovascular disease effect estimates in our study were fairly consistent with no association although some confidence intervals were wide. This may be due in part to low case counts for cardiovascular outcomes, and it is important to note that lack of association in our study does not mean that one does not exist. Overall, there are both fewer wildfire studies examining cardiovascular morbidity, and fewer that found a positive relationship between cardiovascular morbidity and wildfire PM than respiratory morbidity, and the majority of studies focused on PM_10_ rather than PM_2.5_ [6]. Moore, et al. [13] found similar null cardiovascular results in their study of 2003 fires and PM_2.5_ in the Kamloops and Kelowna regions of British Columbia. The authors suggest that wildfire smoke may have a selective effect on respiratory outcomes, thus the lack of association. Alternatively, it is plausible that those who know they are at risk for a cardiovascular event may decide to alter their behavior and stay inside or temporarily relocate from an area expected to be impacted by the wildfires; however, one may also expect that behavior to occur in respiratory outcomes. We would expect this exposure misclassification to result in bias downward. We found positive associations with MI, similar to Rappold, et al. [10], though our associations were only found in 1 h max concentrations, and while the effect estimate was elevated, results were imprecise. However, Rappold, et al. [10] and Delfino, et al. [9], also found evidence for positive associations between exposure to wildfire PM_2.5_ and CHF and all cardiovascular outcomes, respectively. A study conducted in the Denver area during a non-wildfire period generally showed similar results to ours in regard to direction of association between respiratory outcomes and PM_2.5_ concentration. Similar to Kim, et al. [27], we found positive associations between respiratory disease, asthma and PM_2.5_ exposure, with the magnitude of effect increasing with lag time for asthma. Conversely, they found strong associations between cardiovascular disease and PM_2.5_, which were strongest at lag 0, which we did not observe.

Generally, the relationships we observed were weaker when using 1 h max concentrations. This could be because on days when there was a maximum concentration above 30 μg/m^3^, peak concentrations were observed around 7 pm and remained elevated throughout the evening, and these may be times when people are generally inside, thus potentially limiting their exposure. While we were unable to separate the effects of ambient PM_2.5_ from PM_2.5_ stemming from wildfires, it is unlikely that we would normally see ambient concentrations much higher than the first two categorical (up to 20 μg/m^3^ ) groups during a non-wildfire period, as only one PM_2.5_ 24 h maximum concentration was recorded above 35.5 μg/m^3^ in 2012 [28]. Thus, all categorical groups of exposure above the first two are likely to be primarily capturing PM_2.5_ from wildfires.

The short study period limited the number of ED visits available for analysis, which raises concerns about statistical power. Many confidence interval estimates were large, and given this lack of precision we may have been unable to identify modest increases in risk for several of the morbidities. Even so, we did observe positive associations with respiratory disease, asthma/wheeze, and COPD.

We were unable to account for seasonal variations in ED visits, which could have resulted in confounding if the seasonal pattern of ED visits coincided with increases in PM_2.5_ concentrations. However, it is unlikely that seasonal variations in ED visits would fully explain the relationships, as PM_2.5_ concentrations had considerable spatial and temporal variation during the wildfire period, thus potentially obscuring any strong seasonal relationship that might normally exist.

During the Waldo Canyon fire, it was estimated that approximately 32,000 people evacuated the area [3]. It is possible that those who chose to evacuate had medical conditions that would make them more susceptible to PM_2.5_. Many of those who evacuated likely ended up in a different part of Colorado, and if they went to the hospital in a different area, their exposure classification would still be based on their Waldo Canyon area residence, resulting in an exposure misclassification. 91.1 % of emergency department visits and hospitalizations could be geocoded, although it is unlikely that geocoding success was related to PM_2.5_ from wildfires. Similarly, this study was not able to take into account any longer-term effects or adaptations that may have occurred due to the Lower North Fork Fire and Little Sand Fire, which both occurred before June 5^th^ [29, 30]. In an analysis of health effects of the 2003 southern California fires, Kunzli, et al. [31], found that children with asthma were more likely to take preventative measures to reduce exposure and mitigate effects. In addition, when mitigation strategies are used, they have been effective at reducing indoor concentrations [32]. If adaptation due to longer exposures or previous exposures occurred we might expect results that were closer to the null. It is also possible that those exposed to wildfire smoke for long periods of time eventually stop trying to limit their exposure.

We are not able to quantify how the bias toward higher concentrations in the WRF-Chem model could have impacted our estimates, as we are not able to ascertain if exposure bias increased at the same time as daily case count were high. Furthermore, in some cases the model can be shifted in time and/or spatial location, leading to a larger percent different between observed and modelled data. As shown in Fig. 1, areas where the plumes were expected to be has considerably higher PM concentration than areas without the plume, and if the model was slightly off in the location of the plume it may show a large difference in PM concentration compared to the observed data.

## Conclusions

In this study we estimated concentration-response effects of PM_2.5_ over a long-lasting fire period, and our analyses spanned a large geographic area. The study takes into account spatially varying exposure, rather than assigning a uniform exposure during wildfire periods, and also accounts for day to day temporal variability in PM_2.5_. The conditional logistic regression models were able to control for the spatial variations in socio-economic status and population density at the 12 km by 12 km grid level.

People are exposed to wildfire particulate matter relatively infrequently compared to other ambient air pollutants, but there is some evidence to suggest that PM_2.5_ from wildfires may have a stronger adverse effect on respiratory morbidity at the same levels [33], and that there is a difference in toxicological response based on particulate matter source [7, 8, 33]. With climate change, researchers project both longer burn periods and more intense fires, and thus the potential for a greater number of people experiencing adverse health effects due to exposure to wildfire smoke. Furthermore, these smoke plumes may move great distances, impacting people not located near the wildfire itself [34]. Future studies should focus on tracking evacuation and behavior patterns during wildfire periods to help elucidate uncertainties related to exposure measurement error that may be unique to this type of event. While this study, combined with previous toxicological and epidemiologic studies, provides evidence for adverse health effects with exposure to wildfire air pollutants, large gaps in knowledge still exist. This is particularly important when considering that lengthier burn seasons and more intense fire periods are projected for the future [35, 36].

## Abbreviations

CHF, congestive heart failure; COPD, chronic obstructive pulmonary disease; CVD, cardiovascular disease; ED, emergency department; IHD, ischemic heart disease; MI, acute myocardial infarction; OR, odds ratio; PM, particulate matter; WRF- Chem, Weather Research and Forecasting Model with Chemistry

## Additional files

Additional file 1: Figure S1. A Monitor data compared to co-located modelled data. A figure showing the 24 h and hourly (When available) monitor data compared to the modelled data at the same time and location. (PDF 570 kb) Additional file 2: Table S1. A The number of cases per categorical PM. A table of the number of cases for each respiratory and cardiovascular outcome by 24 h mean and 1 h max categorical PM. Each category represents a 10ug/m3 increase in PM2.5 concentration. (DOCX 17 kb)
