# Supplementary material for: The association of wildfire smoke with respiratory and cardiovascular emergency department visits in Colorado in 2012: a case crossover study
Source: Environ Health. 2016 Jun 4;15:64. doi: 10.1186/s12940-016-0146-8 (PMC4893210; doi:10.1186/s12940-016-0146-8)
Supplement: Additional file 1: Figure S1. — A Monitor data compared to co-located modelled data. A figure showing the 24 h and hourly (When available) monitor data compared to the modelled data at the same time and location. (PDF 570 kb) [file 12940_2016_146_MOESM1_ESM.pdf]

Additional file 1

Figure S1. A: Monitor data compared to co-located modelled data

ID: Site ID

DOY: Day of the year

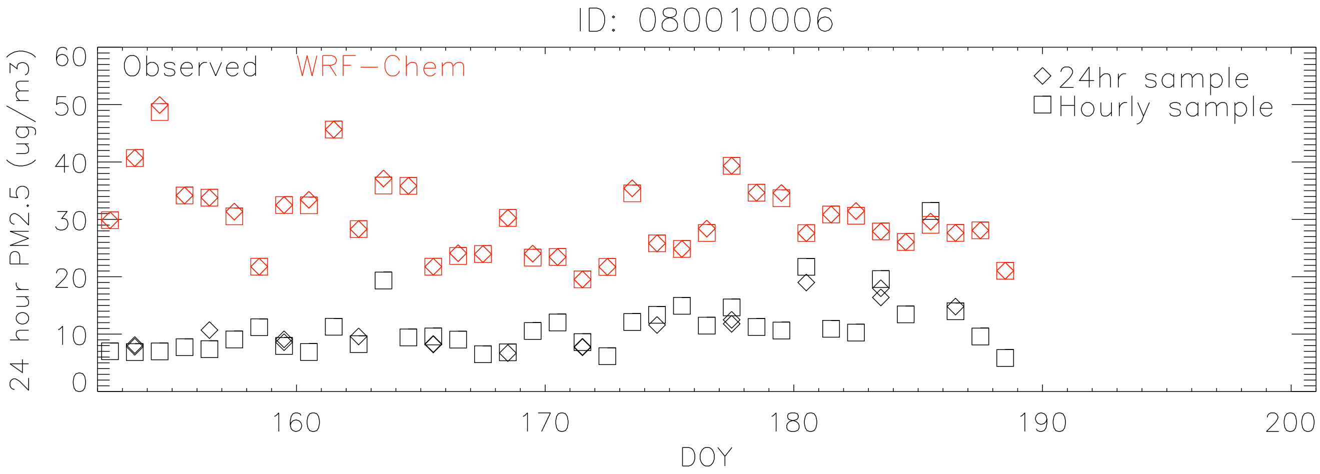

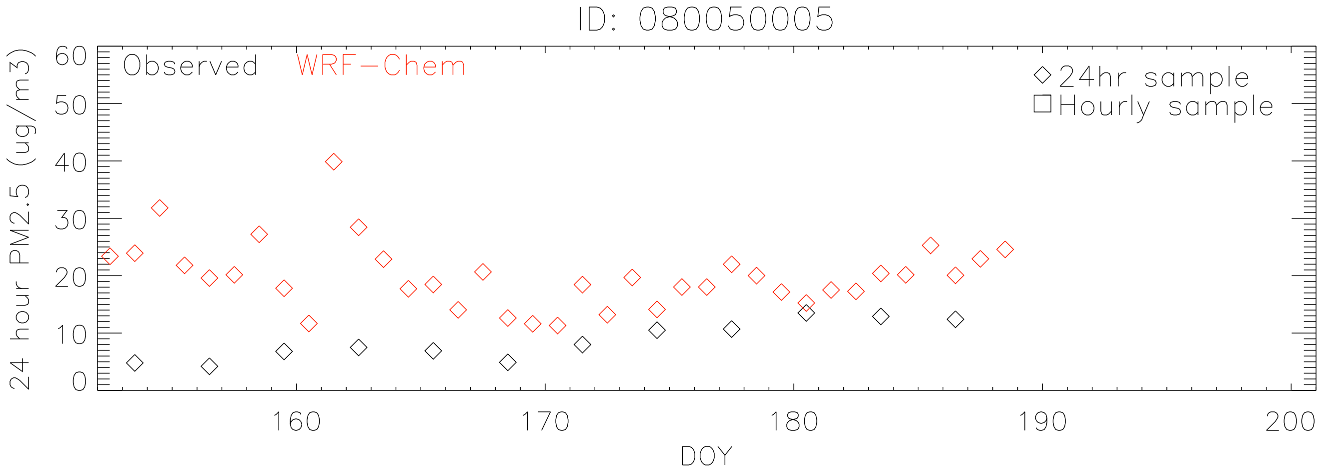

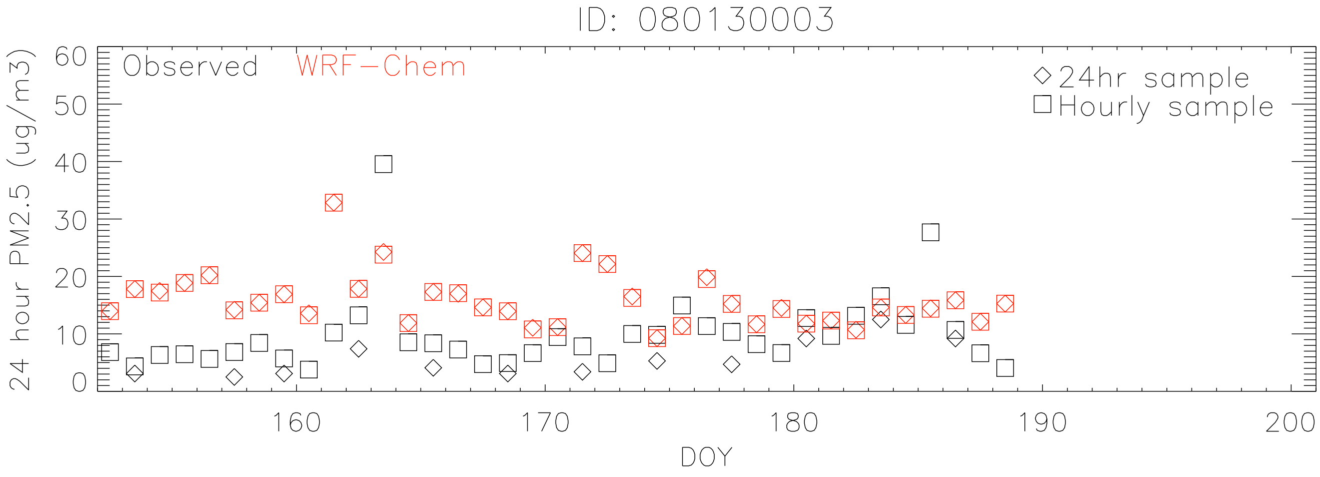

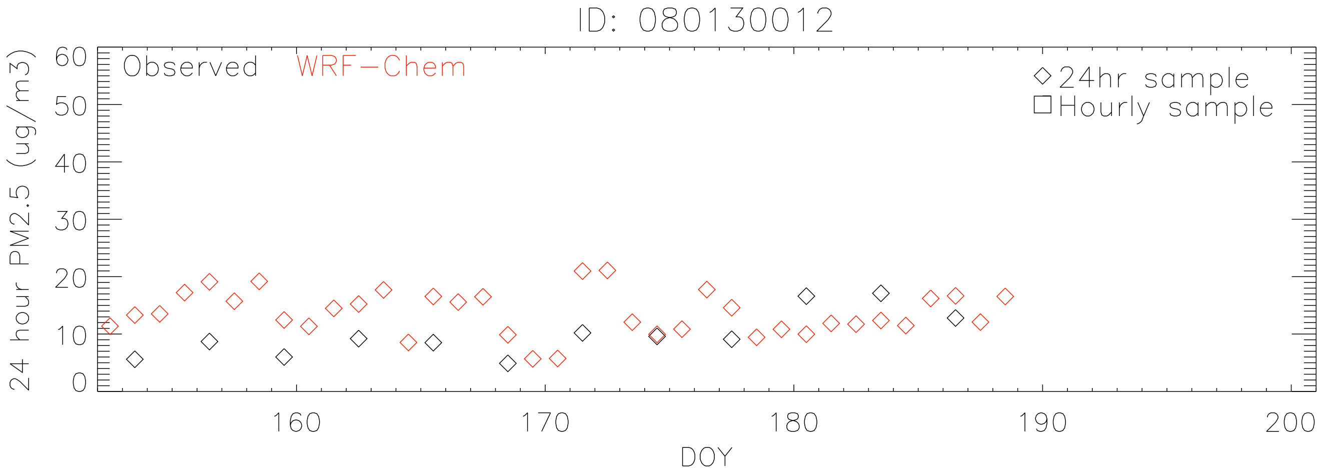
ID: 080130012
Observed
WRF−Chem
24hr sample
Hourly sample
24 hour PM2.5 (ug/m3)
0
10
20
30
40
50
60
160
170
180
190
200
DOY

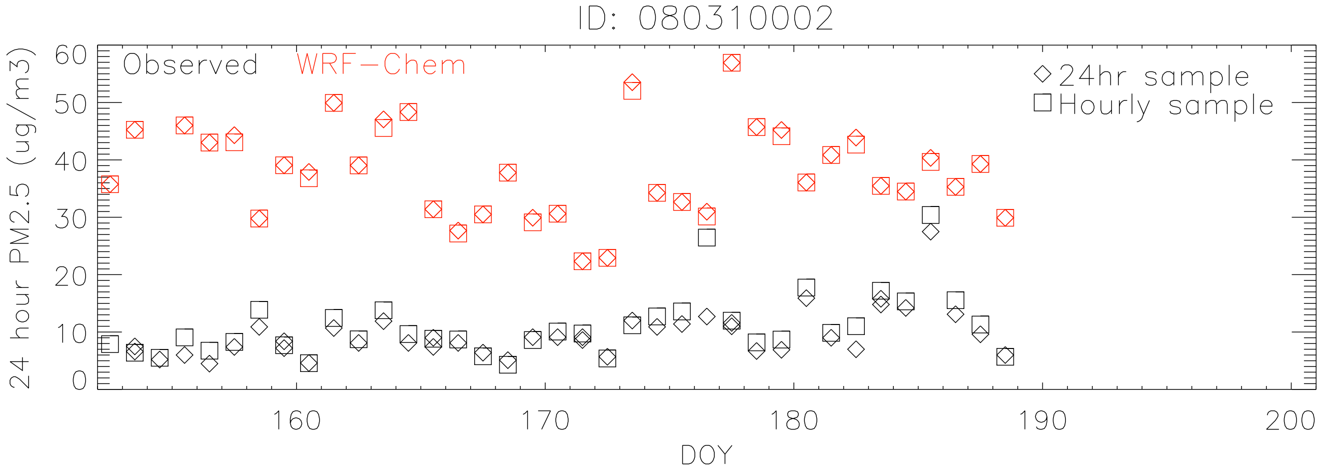
ID: 080310002
Observed
WRF−Chem
24hr sample
Hourly sample
24 hour PM2.5 (ug/m3)
0
10
20
30
40
50
60
160
170
180
190
200
DOY

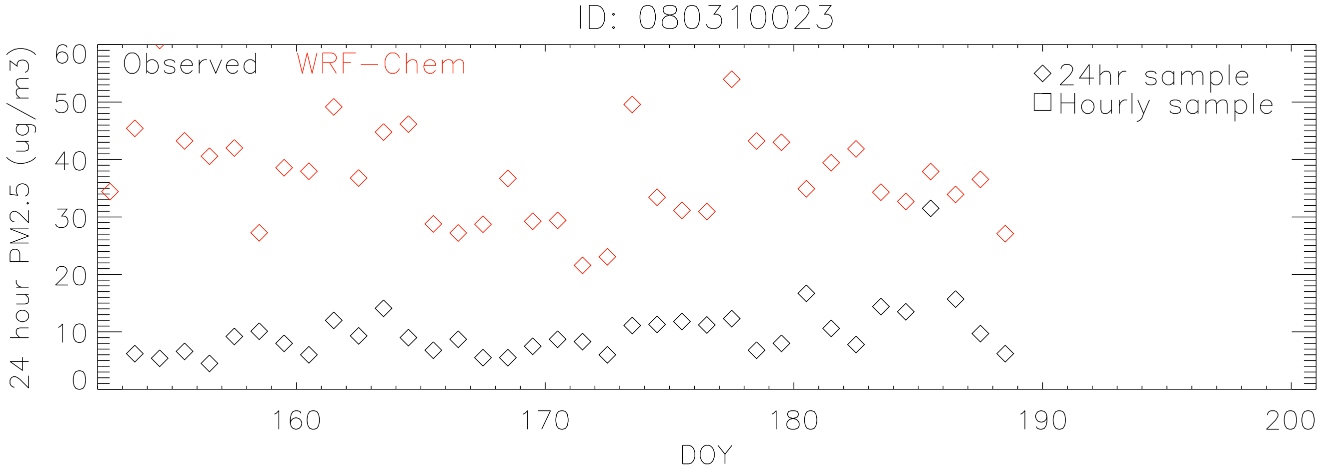
ID: 080310023
Observed
WRF−Chem
24hr sample
Hourly sample
24 hour PM2.5 (ug/m3)
0
10
20
30
40
50
60
160
170
180
190
200
DOY

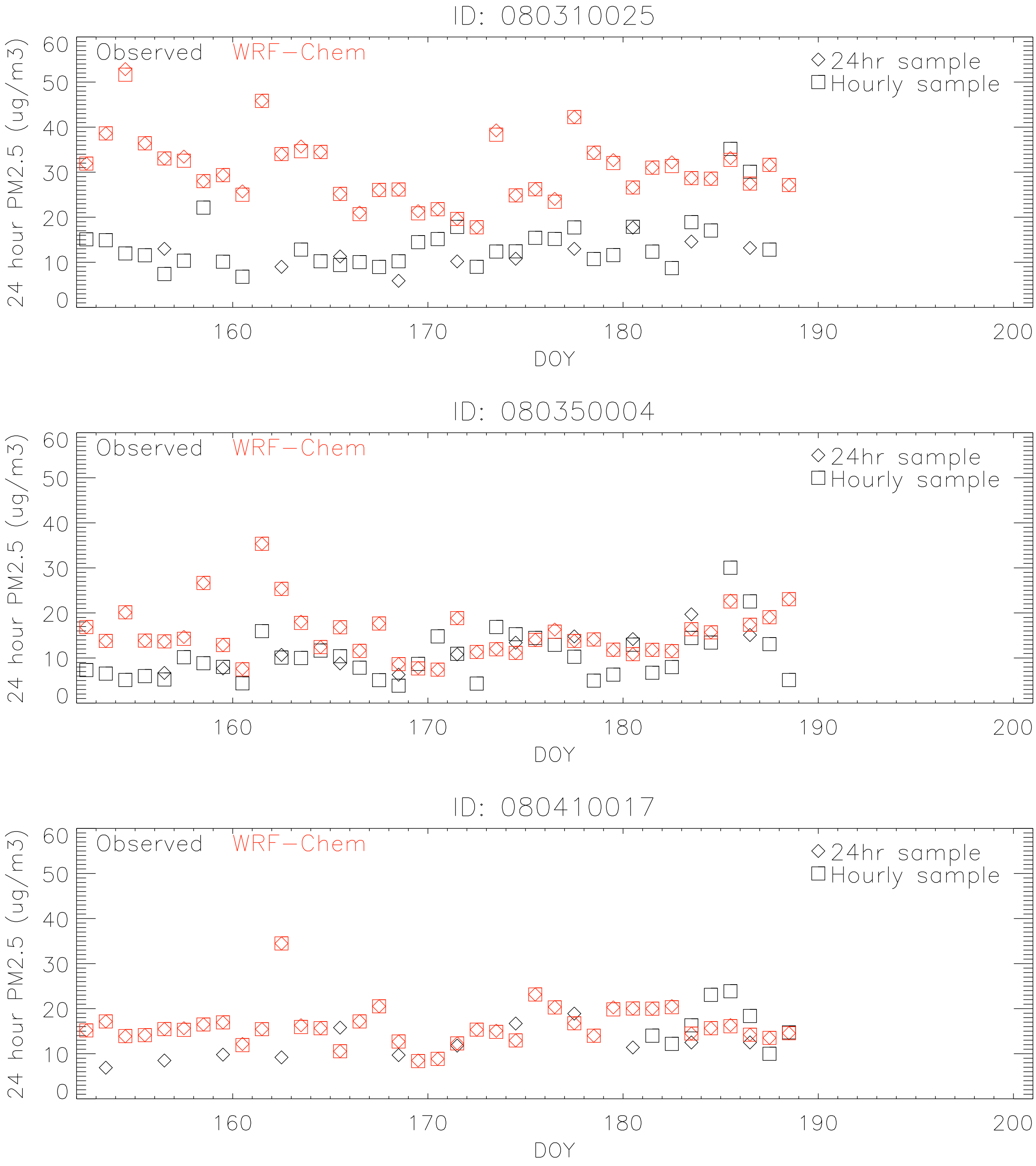
ID: 080310025
Observed WRF−Chem
24hr sample
Hourly sample
24 hour PM2.5 (ug/m3)
DOY
ID: 080350004
Observed WRF−Chem
24hr sample
Hourly sample
24 hour PM2.5 (ug/m3)
DOY
ID: 080410017
Observed WRF−Chem
24hr sample
Hourly sample
24 hour PM2.5 (ug/m3)
DOY

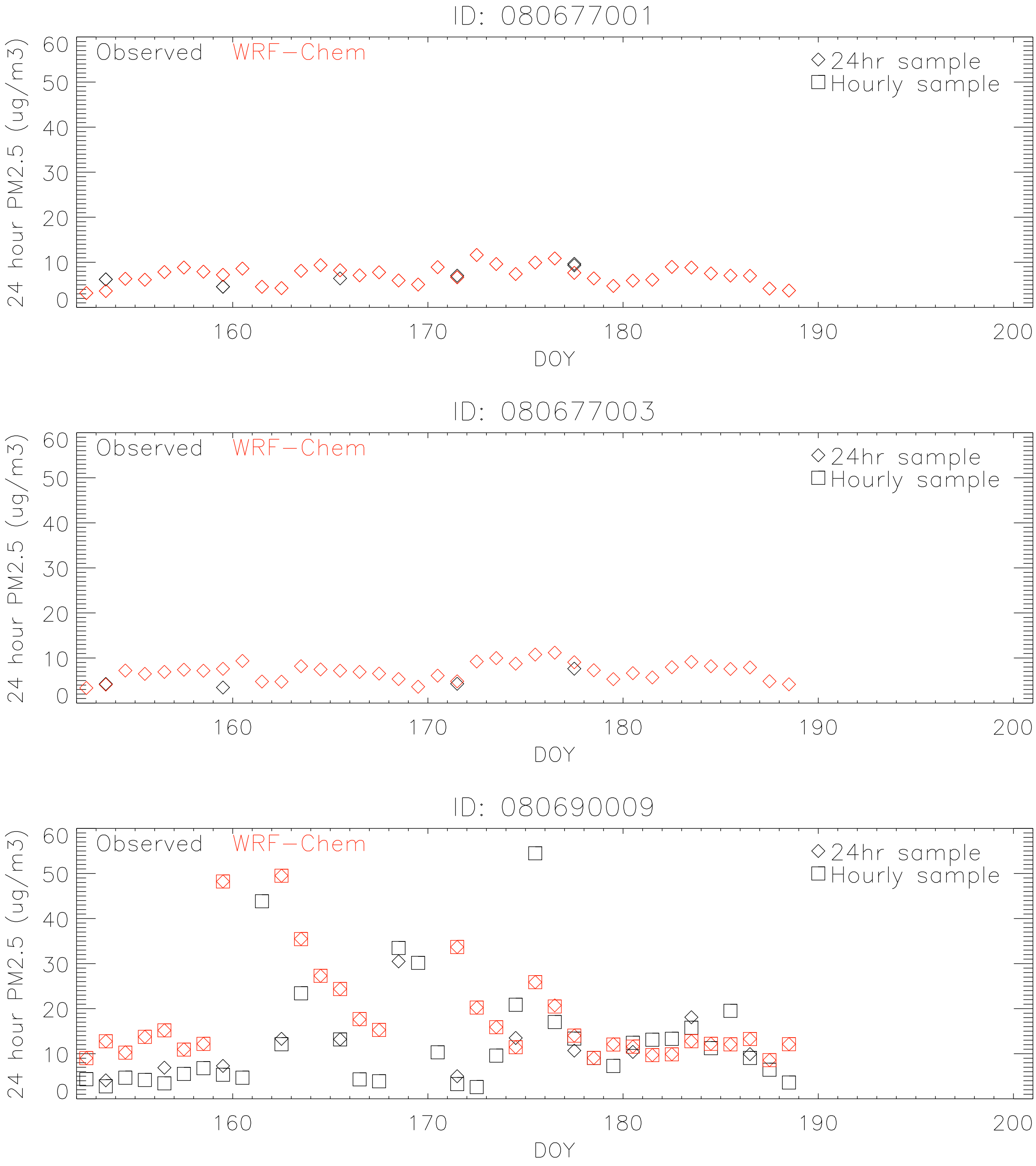
ID: 080677001
Observed
WRF−Chem
24hr sample
Hourly sample
24 hour PM2.5 (ug/m3)
DOY
0
10
20
30
40
50
60
160
170
180
190
200
ID: 080677003
Observed
WRF−Chem
24hr sample
Hourly sample
24 hour PM2.5 (ug/m3)
DOY
ID: 080690009
Observed
WRF−Chem
24hr sample
Hourly sample
24 hour PM2.5 (ug/m3)
DOY

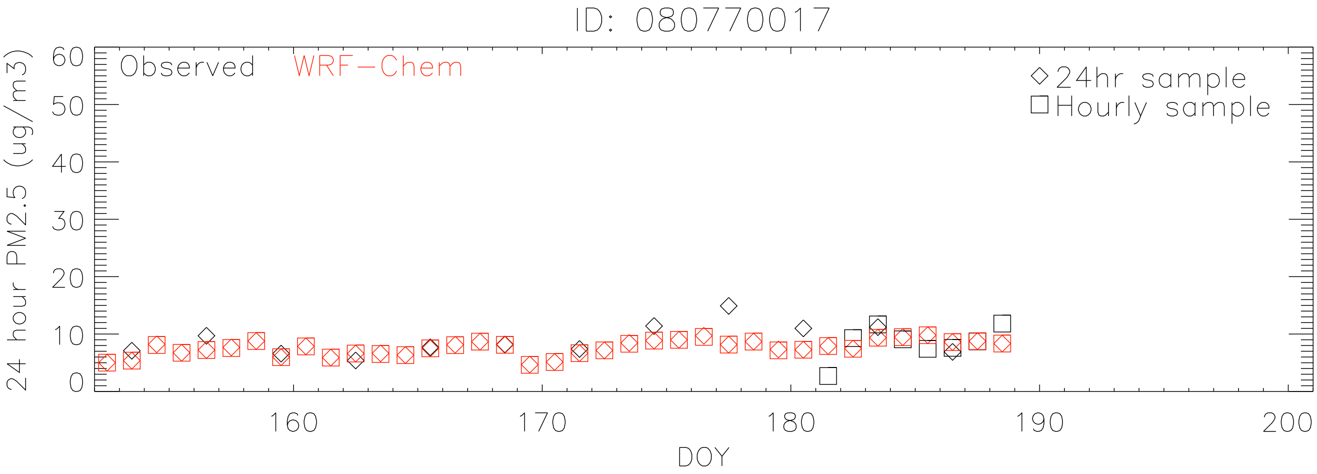
ID: 080770017
Observed
WRF−Chem
24hr sample
Hourly sample
24 hour PM2.5 (ug/m3)
0
10
20
30
40
50
60
160
170
180
190
200
DOY

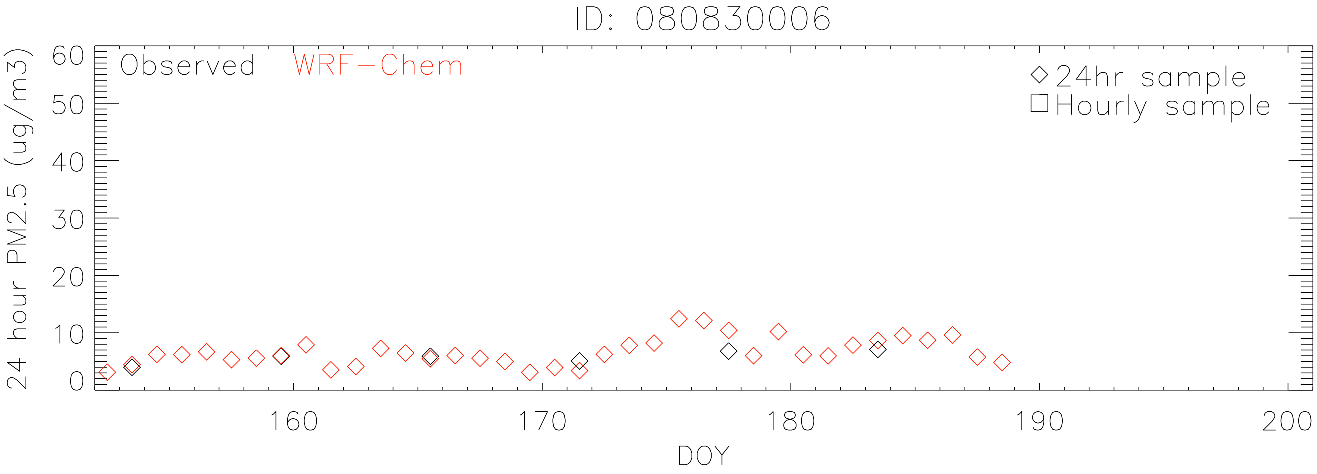
ID: 080830006
Observed
WRF−Chem
24hr sample
Hourly sample
24 hour PM2.5 (ug/m3)
0
10
20
30
40
50
60
160
170
180
190
200
DOY

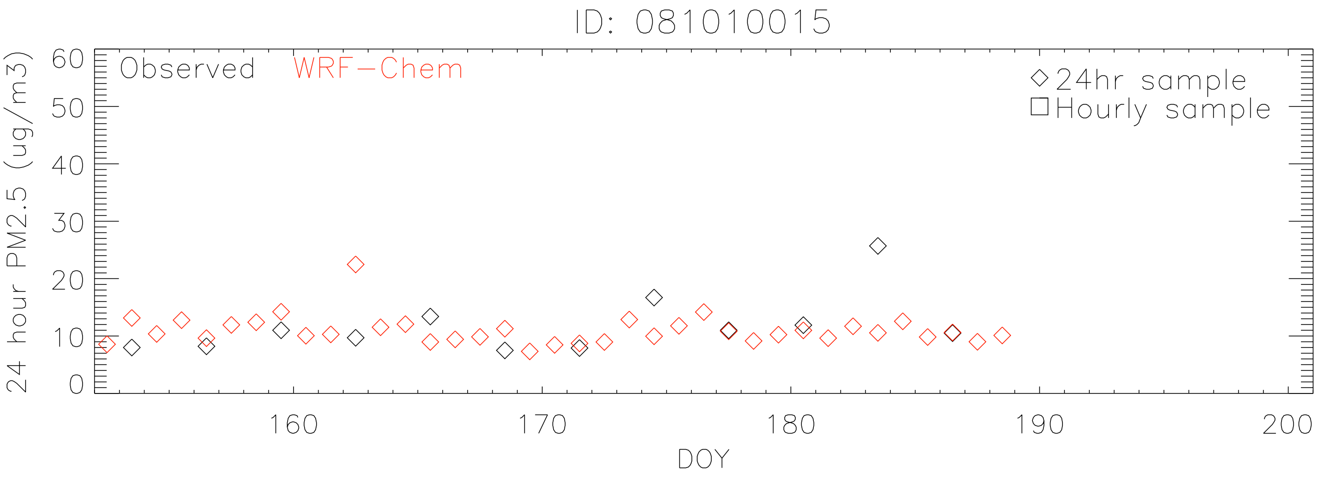
ID: 081010015
Observed
WRF−Chem
24hr sample
Hourly sample
24 hour PM2.5 (ug/m3)
0
10
20
30
40
50
60
160
170
180
190
200
DOY

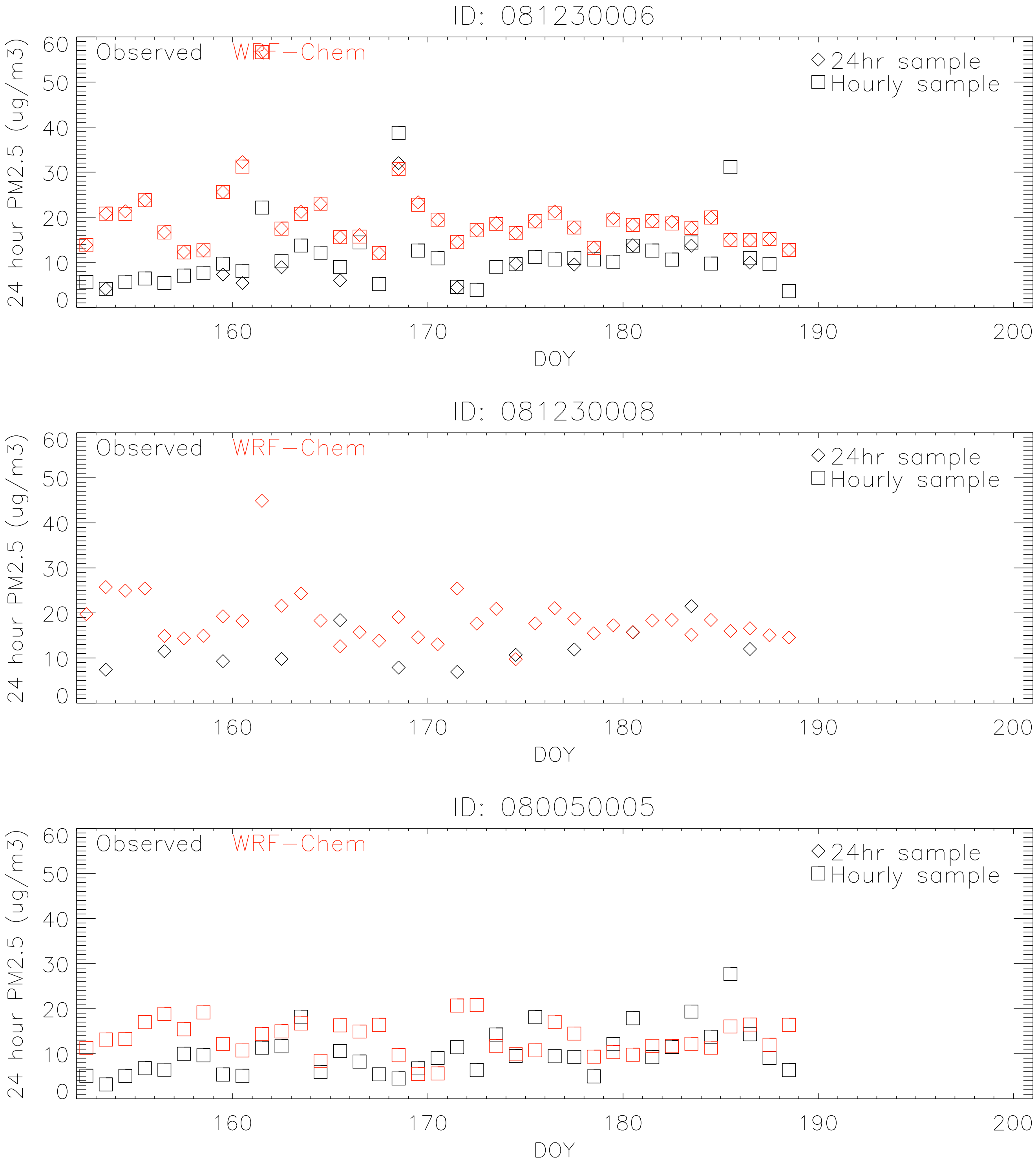
ID: 081230006
Observed
WRF−Chem
◇24hr sample
□Hourly sample
24 hour PM2.5 (ug/m3)
0
10
20
30
40
50
60
160
170
180
190
200
DOY
ID: 081230008
Observed
WRF−Chem
◇24hr sample
□Hourly sample
24 hour PM2.5 (ug/m3)
0
10
20
30
40
50
60
160
170
180
190
200
DOY
ID: 080050005
Observed
WRF−Chem
◇24hr sample
□Hourly sample
24 hour PM2.5 (ug/m3)
0
10
20
30
40
50
60
160
170
180
190
200
DOY

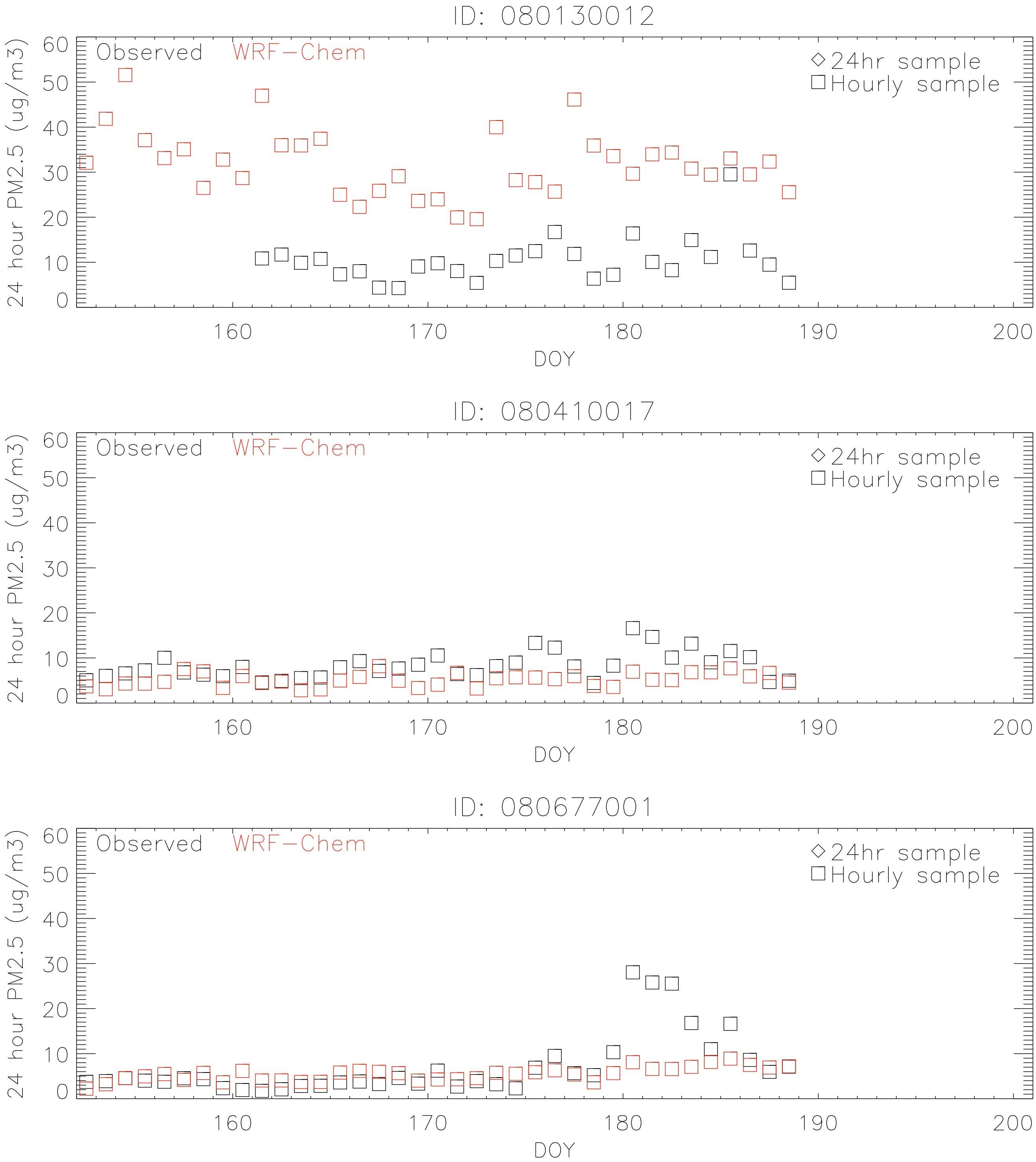
ID: 080130012
ID: 080410017
ID: 080677001
Observed
WRF−Chem
◇24hr sample
□Hourly sample
24 hour PM2.5 (ug/m3)
0
10
20
30
40
50
60
160
170
180
190
200
DOY
