# Supplementary material for: The association of wildfire smoke with respiratory and cardiovascular emergency department visits in Colorado in 2012: a case crossover study
Source: Environ Health. 2016 Jun 4;15:64. doi: 10.1186/s12940-016-0146-8 (PMC4893210; doi:10.1186/s12940-016-0146-8)
Supplement: Additional file 2: Table S1. — A The number of cases per categorical PM. A table of the number of cases for each respiratory and cardiovascular outcome by 24 h mean and 1 h max categorical PM. Each category represents a 10ug/m3 increase in PM2.5 concentration. (DOCX 17 kb) [file 12940_2016_146_MOESM2_ESM.docx]

Additional file 2

Table S1. A The number of cases per categorical PM

|  |  | |  |  |  |  | |  | |  | | |  | | | | | |  |  |
| --- | --- | --- | --- | --- | --- | --- | --- | --- | --- | --- | --- | --- | --- | --- | --- | --- | --- | --- | --- | --- |
| **Health Endpoint** | | **24 hour mean ^*^** | | | | | | | | | **1- hour max ^*^** | | | | | | |  |  |  |
| **Respiratory** | | **0-10** | **10- 20** | **20-30** | | | **30-40** | | **> 40** | | | **0-10** | | **10- 20** | **20-30** | **30-40** | **> 40** |  |  |  |
| Asthma & Wheeze | | 135 | 493 | 312 | | | 82 | | 114 | | | 31 | | 124 | 75 | 176 | 622 |  |  |  |
| Upper respiratory infection | | 493 | 1,455 | 906 | | | 233 | | 289 | | | 120 | | 442 | 505 | 505 | 1 715 |  |  |  |
| Pneumonia | | 216 | 422 | 217 | | | 54 | | 46 | | | 64 | | 173 | 152 | 152 | 392 |  |  |  |
| Bronchitis | | 73 | 205 | 94 | | | 23 | | 18 | | | 17 | | 81 | 64 | 64 | 176 |  |  |  |
| Chronic obstructive pulmonary disease | | 134 | 280 | 112 | | | 49 | | 53 | | | 44 | | 106 | 89 | 89 | 265 |  |  |  |
| Respiratory disease | | 1,058 | 2,906 | 1,668 | | | 453 | | 525 | | | 278 | | 936 | 1,007 | 1,007 | 3,222 |  |  |  |
| **Cardiovascular** | | | |  | | |  | |  | | |  | |  |  |  |  |  |  |  |
| Acute myocardial infarction | | 115 | 215 | 86 | | | 20 | | 26 | | | 35 | | 99 | 96 | 75 | 157 |  |  |  |
| Ischemic heart disease | | 180 | 319 | 150 | | | 33 | | 40 | | | 61 | | 141 | 136 | 123 | 261 |  |  |  |
| Dysrhythmia | | 222 | 492 | 196 | | | 53 | | 37 | | | 68 | | 177 | 219 | 166 | 370 |  |  |  |
| Congestive heart failure | | 123 | 230 | 107 | | | 27 | | 23 | | | 41 | | 591 | 91 | 79 | 198 |  |  |  |
| Ischemic Stroke | | 132 | 256 | 119 | | | 39 | | 30 | | | 40 | | 107 | 105 | 90 | 234 |  |  |  |
| Peripheral vascular disease | | 83 | 190 | 89 | | | 24 | | 25 | | | 30 | | 65 | 84 | 67 | 165 |  |  |  |
| Cardiovascular disease | | 740 | 1,487 | 661 | | | 176 | | 155 | | | 240 | | 591 | 635 | 525 | 1,228 |  |  |  |
| *Each category is an increase in 10 μg/m^3^ | |  |  |  | | |  | |  | | |  | |  |  |  |  |  |  |  |
